# Report of Two Cases of Labor Following Ventral Fixation

**Published:** 1902-02

**Authors:** L. G. Hanley

**Affiliations:** Buffalo, N. Y.; Gynecologist to the Buffalo Hospital of the Sisters of Charity


### CLINICAL REPORT.

### Report of Two Cases of Labor Following Ventral Fixation.

By L. G. HANLEY, M. D., Buffalo, N. Y.,
Gynecologist to the Buffalo Hospital of the Sisters of Charity.

Case I.—Mrs. B., age 30, mother of two children, had one abortion in 1897, was admitted to hospital April 12, 1898; age at first menstruation, 14; menstruation prior to marriage normal; menstruation after marriage irregular, accompanied by considerable pain, severe dysmenorrhea; family history good. When first child was born, was confined to her bed for two months (as she says, with child-bed fever). When admitted to hospital, complained of severe pain in back and over region of left ovary. There was considerable leucorrheal discharge. Examination revealed a mass on left side of pelvis—pyosalpinx?

There is a retroversion of the uterus, with prolapse. Patient prepared for abdominal section. On opening the abdominal cavity there was found a large pyosalpinx on left side, with uterus bound down by adhesions. Tube and ovary on left side removed, adhesions broken up and uterus anchored to anterior abdominal wall.

The method used was the following, which has been my method on several occasions: peritoneum stripped off for about one inch on either side of incision; serosa of uterus nearly corresponding with the size of peritoneum, stripped off from each side of uterus. Raw surface of peritoneum and raw surface of serosa are brought in apposition, and united by a few stitches of fine catgut, but not until a braided silk suture has been passed through the skin, fascia, muscle, but not the peritoneum, extending from one cornu of uterus under the serosa to the other cornu and going through on the other side of incision, through the muscle, fascia and skin. A silkworm-gut suture was passed anteriorly, going through skin, fascia, muscle and peritoneum, another posteriorly going through skin, fascia and peritoneum; these, together with the braided silk suture, closed the abdominal wound.

Patient left hospital in three weeks. Was examined by me one year after, when uterus was found to be in normal position. Menstrual function normal.

May 31, 1901, was delivered by me of a 7½ pound boy; labor normal in every respect; vertex presentation, position L. O. A.

Case II.—Mrs. S., age 29; admitted to hospital May 15, 1900; number of children, 2; number of abortions, 2; was curetted in January, 1900, having had an abortion; complained of pain in the back and abdomen and flows profusely at menstrual periods; great pain at defecation. Examination revealed incomplete laceration of perineum and a large swelling in left side of pelvis; also slight prolapse with retroversion.

Patient prepared for operation. On opening the abdominal cavity a large hematosalpinx was discovered in left side, which was removed. The uterus was anchored to the anterior abdominal wall by the same method as described in previous case. Specimen was 4½ inches long by 3 deep. Perineum was repaired. Left hospital in twenty days.

September 24, 1901, was attended by me during confinement; labor normal; vertex presentation; position L. O. A. Cervix seemed the same as in any other case of normal labor—was not retracted beyond the ordinary and patient made a good recovery. Baby weighed 6 pounds 2 ounces. Uterus still in good position.

I report these two cases, as they are the only ones out of a record of fifty-three of ventral fixation, that have been delivered by me. I have learned that some of the cases operated on by me have borne children after operation, but can only speak definitely of the two here presented.

